# The influence of dehydration on the prognosis of acute ischemic stroke for patients treated with tissue plasminogen activator

**DOI:** 10.1186/s12872-017-0590-6

**Published:** 2017-06-13

**Authors:** Fei-Fan Wu, Yen-Chu Hung, Y. H. Tsai, Jen-Tsung Yang, Tsong-Hai Lee, Chia-Wei Liow, Jiann-Der Lee, Chung-Jen Lin, Tsung-I Peng, Leng-Chieh Lin

**Affiliations:** 1 Department of Emergency Medicine, Chang Gung Memorial Hospital, No. 6, W. Sec., Jiapu Rd., Puzih City, Chiayi County 613 Taiwan, ROC; 2 Department of Neurology, Chang Gung University, Taoyuan, Taiwan; 3 Department of Diagnostic Radiology, Chang Gung University, Taoyuan, Taiwan; 4 Division of Neurosurgery, Department of Surgery, Chang Gung Memorial Hospital, Chiayi, and College of Medicine, Chang Gung University, Taoyuan, Taiwan; 5 Department of Nursing, Chang Gung University of Science and Technology, Chiayi Campus, Chiayi, Taiwan; 6 Stroke Center and Department of Neurology, Linkou Chang Gung Memorial Hospital, and College of Medicine, Chang Gung University, Taoyuan, Taiwan; 7 Department of Neurology, Kaohsiung Chang Gung Memorial Hospital, and College of Medicine, Chang Gung University, Kaohsiung, Taiwan; 8 Department of Neurology, Keelung Chang Gung Memorial Hospital, and College of Medicine, Chang Gung University, Keelung, Taiwan

**Keywords:** Ischemic stroke, Modified Rankin scale, Barthel index, Dehydration, BUN/Cr, Stroke registry

## Abstract

**Background:**

Many studies have determined that dehydration is an independent predictor of outcome after ischemic stroke (IS); however, none have determined if the use of thrombolytic therapy modifies the negative impact of poor hydration. To inform the stroke registry established at our institution, we conducted a retrospective study to determine if dehydration remains a negative prognostic factor after IS patients treated with tissue plasminogen activator (tPA).

**Methods:**

Between 2007 and 2012, we recruited 382 subjects; 346 had data available and were divided into 2 groups on the basis of their blood urea nitrogen/creatinine (BUN/Cr) ratio. Dehydrated subjects had a BUN/Cr ratio ≥ 15; hydrated subjects had a BUN/Cr < 15. The primary outcome was impairment at discharge as graded by the Barthel Index (BI) and the modified Rankin Scale (mRS).

**Results:**

The dehydration group had a greater mean age; more women; lower mean levels of hemoglobin, triglycerides, and sodium; and higher mean potassium and glucose levels. A favorable outcome as assessed by the mRS (≤2) was significantly less frequent among dehydrated subjects, but a favorable outcome by the BI (≥60) was not. Logistic regression and multivariate models confirmed that dehydration is an independent predictor of poor outcome by both the mRS and the BI; however, it was not predictive when patients were stratified by Trial of Org 10,172 in Acute Stroke Treatment subtype.

**Conclusions:**

Our findings indicate that use of thrombolytic therapy does not eliminate the need to closely monitor hydration status in patients with IS.

## Background

Ischemic stroke (IS) is a global problem that is associated with significant mortality, decreased lifespan, and cost. While analyses of data from The World Health Organization’s (WHO) Global Burden of Disease Study indicate that both the worldwide incidence of IS and the associated mortality have decreased over the last few decades [1, 2], they have also revealed that IS remains one of the top causes of mortality worldwide, leading to similar number of deaths as respiratory tract infections and chronic obstructive pulmonary disease [3]. Moreover, IS has a significant impact on years lived with a disability (YLD), and IS-associated disability has increased worldwide between 1990 and 2013 [4]. In Taiwan, stroke is the second leading cause of death [5], with spending recently estimated at almost $400 million dollars annually [6]. Among stroke subtypes, IS accounts for almost three-quarters of all strokes [7].

Extensive literature exists suggesting a diversity of factors are associated with an unfavorable outcome after acute IS. Clinical characteristics such as severity [8, 9], age at stroke onset [10, 11], gender [12], and existence of comorbid conditions [13, 14] are well established factors. However, evidence now indicates that hydration status can also have a significant impact on outcome. In 2000, Bhalla and colleagues conducted one of the first investigations of the effects of dehydration on outcomes after IS and found that 3-month survivors had lower plasma osmolality than did non- survivors [15]. This finding was supported by Kelly et al., who in 2004 reported that dehydration was associated with a higher risk of venous thromboembolism [16]. More recently, Rowat et al. and Schrock et al. assessed mortality and discharge status [17, 18]. At our stroke referral center in Taiwan, a registry established in 2006 (Stroke Registry of the Chang Gung Healthcare System [SRICHS] [19]) has allowed a comprehensive assessment of the negative impact of dehydration on numerous features of IS progression and recovery, including neurological deterioration, development of collaterals, infection rate, and performance on disability scales [20–26]. Collectively, the data suggest that hydration therapy should be a central feature of stroke management, and recent guidelines acknowledge this [27].

Despite the fact that dehydration should now be accepted as a risk factor for poor outcomes after stroke, we know very little of how its negative effects are attenuated or modified by current treatments of IS, particularly thrombolytic therapy with tissue plasminogen activator (tPA). While tPA is associated with an increased risk of hemorrhage in patients with IS [28], current guidelines highlight the efficacy of tPA in dissolving blood clots and stress the importance of immediate thrombolytic therapy for eligible patients [27]. These may even include patients with a history of antiplatelet therapy at the time of the stroke [29]. Though not yet proven, it seems likely that dehydration may lead to poor outcomes because of an increased rate of thromboembolisms. In this scenario, thrombolytic therapy would offset the negative effects of dehydration. Currently, data are lacking that describe the effects of tPA treatment for dehydrated patients after IS. To fill this gap in knowledge, this study was conducted to determine if dehydration remains a poor prognostic factor among patients who were treated with tPA for an acute IS. We assessed outcome with the Barthel Index (BI) and the modified Rankin Scale (mRS) [30, 31]. The mRS is a general scale that describes a patient’s disability along a continuum, from no symptoms to severely disabled, with grades of 0 to 5. The BI assesses a patient’s ability to perform 10 common tasks of daily living, with scores ranging from 0 to 100 on the basis of the level of independence with which the task is performed. Each scale has unique strengths and weaknesses, with reliability and sensitivity being the biggest issue with the mRS and BI, respectively [32]. Despite these limitations, they are the two most commonly used scales in stroke trials [33, 34].

## Methods

### The stroke registry of the Chang Gung healthcare system

This retrospective study assessed patient data collected prospectively from the Stroke Registry of the Chang Gung Healthcare System (SRICHS) [19]. All data had already been collected and was in the registry prior to the start of the current study. SRICHS is an electronic chart-based stroke registry system that was established on 1 March 2007. To improve the quality of stroke care, all data from the registry are incorporated into the electronic medical chart system of the Chang Gung Medical System, as with other registry studies [18], and are monitored regularly by consensus conferences attended by the staff of the neurology and computer science departments. All patients with an International Classification of Diseases 9 (ICD 9) 430437 classification indicating ischemic or hemorrhagic stroke are automatically recruited into this registry system. Clinical data are recorded by primary care medical staff, and any numerical data including laboratory tests and carotid Doppler flow velocity are automatically downloaded from the hospital information system to the stroke registry system to eliminate human error during data entry. The information is further proof-read by stroke center staff to ensure the accuracy of the registered data.

### Patient recruitment, procedures, and data collection

This study was approved by the Human Studies Institutional Review Board at the Chia-Yi Chang Gung Memorial Hospital. Between January 2007 and December 2012, patients who experienced an acute ischemic stroke and received tPA were recruited for analysis at the hospital. Levels of blood urea nitrogen (BUN) and/or creatinine (Cr) may not have been examined, as Chia-Yi Chang Gung Memorial Hospital is a tertiary medical center and levels might have been measured in local hospitals. Accordingly, patients whose BUN and/or Cr tests were not conducted in our hospital were not included in the analysis. The eligible patients were divided into 2 groups based on hydration status, as assessed by the BUN/Cr ratio. This ratio has been used as an indicator of dehydration in previous studies by our group and by others [17, 18, 20–26, 35–37]. Dehydrated patients had a BUN/Cr ratio ≥ 15 and those patients with a BUN/Cr ratio < 15 were placed in the non-dehydrated group. A normal BUN/Creatinine ratio ranges from 10 to 15 [38, 39]; therefore, we defined dehydration as a BUN/Creatinine ratio ≥ 15. This ratio was preferred by other groups for the same reason [18, 36, 37]. The samples used to determine the Bun/Creatinine ratio were collected at the time of admission; therefore, dehydration status was assessed within 3 h of stroke onset.

The study inclusion criteria were as follows: the patient received a clinical diagnosis of ischemic stroke causing neurological deficit, and the time from symptom onset to admission was <3 h. Exclusion criteria were the following: 1) intracranial hemorrhage on CT; 2) a clinical presentation suggestive of subarachnoid hemorrhage; 3) neurologic surgery, serious head trauma, or a previous stroke in the past 3 months; 4) uncontrolled hypertension (>185 mmHg systolic blood pressure [SBP] or >110 mmHg diastolic blood pressure [DBP]); 5) a history of intracranial hemorrhage; 6) seizure at stroke onset; 7) known arteriovenous malformation, neoplasm, or aneurysm; 8) active internal bleeding; 9) suspected or confirmed endocarditis; 10) known bleeding diathesis, including: a) a platelet count <100,000, b) heparin within 48 h and an elevated activated partial thromboplastin time (aPTT) greater than the upper limit of normal for the laboratory, c) current use of oral anticoagulants (ex: warfarin) and an international normalized ratio (INR) >1.7, and d) current use of direct thrombin inhibitors or direct factor Xa inhibitors; and 11) an abnormal blood glucose level (<50 or >400 mg/dL). Patients received tPA in accordance with the following formula: body weight (kg) * 0.9 = dose (mg); 10% of the total calculated dose was administered via an intravenous (IV) push over at least 1 min. The remaining 90% of the total calculated dose was administered via IV infusion over 1 h. The mRS and BI were administered at discharge by a general physician or nurse practicioner, who evaluated the symptoms of each patient. The evaluation was then confirmed by a neurology specialist. After the evaluation was confirmed, the scores were recorded in the electronic registry system.

We documented patients’ demographic data, length of hospitalization, and the presence of stroke risk factors including age, sex, levels of BUN and Cr on admission, history of first-ever and recurrent stroke, admission score of the National Institutes of Health Stroke Scale (NIHSS), presence of infections and dysphagia at admission, diagnosis of symptomatic deep vein thrombosis (DVT), and history of hypertension (HTN) and diabetes mellitus (DM). The total length of hospitalization varied substantially among the patients, therefore; outcomes were not assessed at a fixed time interval. HTN was defined as known hypertension before admission or blood pressure ≥ 140/90 mmHg during admission [40]. DM was defined as fasting glucose ≥126 mg/dl, casual plasma glucose ≥200 mg/dl, or glycosylated hemoglobin ≥6.5%. Types of infection monitored at admission included bronchitis, cellulitis, cholangitis, cholecystitis, colitis, conjunctivitis, cystitis, dermatitis, endocarditis, empyema, gastroenteritis, gingivitis, hepatitis, keratitis, mastoiditis, nephritis, otitis, periodontitis, pneumonia, pulpitis, sepsis, sinusitis, spondylitis, synovitis, and infections of the central nervous system, soft tissues, teeth and gums, upper respiratory tract, and urinary tract. Length of hospital stay included the total time of medical care in the emergency department, neurological intensive care unit, and the ordinary ward.

The primary outcome was determination of neurological and functional impairment at discharge as graded by the Barthel Index (BI) and the modified Rankin Scale (mRS). When being discharged, each patient was evaluated with both scales, regardless of hydration status. The secondary outcomes were the identification of factors associated with a favorable prognosis, as assessed by the mRS and the BI, and identification of factors associated with dehydration. A favorable prognosis was a mRS score ≤ 2 or a BI score > 60. We chose a BI score > 60 to represent a favorable outcome because this cut-off value has historically been associated with improvement beyond complete dependence [34, 41]. For the purposes of this study, it was judged to be most informative to have a favorable prognosis include all patients who were no longer completely dependent, as opposed to including only those patients who were mildly dependent or independent.

### Statistical analysis

Continuous variables with a normal distribution are presented as mean and standard deviations (SDs), those with a skewed distribution as median and inter-quartile ranges (IQRs), and categorical variables as counts and percentages (%). Between – group differences were compared with the independent t-test for uniform continuous variables, the Mann–Whitney U test for skewed continuous variables, and the chi-square test for categorical variables. Multivariable logistic regression with the forward stepwise method was performed to examine which factors were associated with a good prognosis. Factors which were significantly different between the good and bad prognosis groups were included in the multivariable model. Factors associated with prognosis were stratified by stroke subtypes of the Trial of Org 10,172 in Acute Stroke Treatment (TOAST) to determine which clinical factors are associated with dehydration for each type of stroke [42]. Statistical analyses were performed by IBM SPSS statistical software version 22 for Windows (IBM Corp, Armond, NY, USA). A two-tailed *P* < 0.05 indicated statistical significance.

## Results

### Characteristics of subjects with and without dehydration

A total of 382 subjects were included in this study. Data for the BUN/Cr ratio were missing for 36 of these subjects; accordingly, the remaining 346 subjects were divided into 2 groups. The 164 subjects without dehydration had a BUN/Cr ratio < 15 while the 182 subjects with dehydration had a BUN/Cr ratio > 15. Complications included infection, hemorrhage, and gout (acute gouty arthritis). The length of hospitalization ranged from 1 to 73 days for patients in the non-dehydration group and 0 to 123 days for those in the dehydration group. Our analysis determined that the subjects in the 2 groups differed significantly for several clinical variables. As shown in Table 1, subjects who were dehydrated were older, more likely to be female, and had higher levels of BUN, glucose, Hb1, and potassium than did those who were not dehydrated (age, *p* = 0.013; female gender, *p* < 0.001; BUN, *p* < 0.001; glucose, *p* = 0.035; Hb1, *p* = 0.02; K, *p* = 0.005). Conversely, well hydrated subjects weighed more and had higher levels of triglycerides (TG), creatinine (Cr), and sodium (Na) than did dehydrated subjects (weight, *p* = 0.023; TG, *p* = 0.038; Cr, *p* < 0.001; Na, *p* = 0.029). The percentage of subjects with a favorable score on the mRS (≤2) was significantly lower in the dehydration group (*p* = 0.012); however, the dehydration and non-dehydration groups had similar proportions of subjects with a favorable BI score (≥60). No significant differences were found between subjects with and without dehydration in the other variables that were assessed (*p* > 0.05; Table 1).

**Table 1:** Characteristics of subjects with and without dehydration

	Non-dehydration (BUN/Cr < 15) (*N* = 164)	Dehydration (BUN/Cr ≥ 15) (*N* = 182)	*p*-value
Age	63.39 ± 13.63	66.87 ± 12.39	0.013^*^
Sex			
Female	41(25%)	84(46.15%)	<0.001^*^
Male	123(75%)	98(53.85%)	
Length of hospitalization	12.2(8–20)	13(8–23)	0.361
BMI (kg/m^2^)	24.56 ± 4.23	24.03 ± 3.70	0.225
GCS	15(11–15)	15(11–15)	0.222
WBC	7.9(6.4–10.05)	7.45(6.3–9.45)	0.207
RBC	4.72(4.34–5.08)	4.65(4.29–4.98)	0.274
Hb	14.18 ± 1.82	13.87 ± 1.83	0.120
Hct	41.8 ± 4.8	41.13 ± 4.56	0.192
PL	209(165–247)	203.5(170–235)	0.825
PT	10.8(10.3–11.6)	11(10.4–11.5)	0.357
APTT	26(24.4–27.9)	25.65(24.3–27.3)	0.380
ALB	3.7(3.4–4.09)	3.78(3.4–4.09)	0.595
LDL	114.5(95–131)	110(91–137)	0.623
HDL	42(36–51)	42.55(36–51)	0.474
TCHO	181(159–204)	178.5(151–208)	0.366
TG	106.5(81.5–153)	94(75–137)	0.038^*^
BUN	11.2(9–13.8)	17(14–21.4)	<0.001^*^
UA	5.71 ± 1.63	5.35 ± 1.67	0.083
AST	26(20–33)	27(20–36)	0.824
ALT	22(17–32)	23(18–32)	0.740
Cr	1(0.87–1.2)	0.87(0.72–1.07)	<0.001^*^
Glucose	123(100–157)	130(111–169.5)	0.035^*^
Hb1	5.9(5.6–6.4)	6(5.7–7.4)	0.020^*^
CRP	12.2(4.9–51.2)	16.97(5.5–61.16)	0.462
Na	139(137.9–141)	138.6(137–140)	0.029^*^
K	3.69(3.4–3.9)	3.71(3.53–4)	0.005^*^
Lymphocyte	29.68 ± 10.37	30.04 ± 12.09	0.771
SPG	1.01(1.01–1.02)	1.02(1.01–1.02)	0.412
SBP	149.5(136–162)	146(130–158)	0.082
NIHSS-Entry	11(7–18)	12.5(7–18)	0.898
NIHSS-Discharge	6(2–12.5)	7(3–15)	0.106
Complication			
Infection			
No	120(73.17%)	123(67.58%)	0.256
Yes	44(26.83%)	59(32.42%)	
Hemorrhage			
No	153(93.29%)	170(93.41%)	0.966
Yes	11(6.71%)	12(6.59%)	
Gout			
No	151(92.07%)	175(96.15%)	0.104
Yes	13(7.93%)	7(3.85%)	
Proteinuria			
No	80(57.55%)	95(60.13%)	0.653
Yes	59(42.45%)	63(39.87%)	
Toast Dx			
Cardioembolism	51(31.1%)	69(37.91%)	0.709
Lacune	27(16.46%)	32(17.58%)	
Large-artery atherosclerosis	40(24.39%)	35(19.23%)	
Other determined	1(0.61%)	2(1.1%)	
Undetermined	38(23.17%)	38(20.88%)	
Favorable prognosis			
mRS ≤ 2	64(39.02%)	48(26.37%)	0.012^*^
BI ≥60	82(50.0%)	75(41.21%)	0.101

Data for the BUN/Cr ratio are missing for 36 subjects

Abbreviations are presented in the list of abbreviations

*p < 0.05, significantly different between dehydration and non-dehydration

### Analysis of prognosis at discharge

To determine if subjects who were dehydrated had a worse prognosis, we used the mRS and the BI to measure disability at the time of discharge. The results of our analysis are shown in Table 2. When subjects were evaluated with the mRS, those who were dehydrated were more likely to have a bad prognosis, defined as mRS > 2 (134 of 250 subjects vs. 48 of 132 subjects with good prognosis; *p* = 0.012). However, when the BI was used to gauge prognosis (BI <60 = bad prognosis), no difference was found (107 of 198 subjects vs. 75 of 184 subjects with good prognosis; *p* = 0.101).

**Table 2:** Comparisons of clinical variables of subjects with a favorable or unfavorable prognosis

	mRS			BI		
Variable	Unfavorable prognosis (*N* = 250)	Favorable prognosis (*N* = 132)	*p*-value	Unfavorable prognosis (*N* = 198)	Favorable prognosis (*N* = 184)	*p*-value
Age	66.62 ± 13.11	61.7 ± 12.4	<0.001^*^	66.84 ± 13.33	62.85 ± 12.48	0.003^*^
Sex			0.100			0.004^*^
Female	99(39.6%)	41(31.06%)		86(43.43%)	54(29.35%)	
Male	151(60.4%)	91(68.94%)		112(56.57%)	130(70.65%)	
Days of admission	15.5(9–24)	9(6.41–13)	<0.001^*^	16.83(10–26)	9.31(7–14)	<0.001^*^
BMI (kg/m^2^)	24.25 ± 3.94	24.34 ± 3.96	0.837	24.33 ± 4.04	24.24 ± 3.85	0.834
GCS	12.95 ± 2.62	13.94 ± 1.94	<0.001^*^	ND	ND	ND
WBC	7.6(6.2–9.6)	8(6.6–10)	0.109	ND	ND	ND
RBC	4.69(4.32–5)	4.67(4.29–5.05)	0.803	4.7(4.29–5.01)	4.68(4.33–5.02)	0.741
Hb	14.01 ± 1.76	14.07 ± 1.92	0.771	ND	ND	ND
Hct	41.51 ± 4.53	41.45 ± 4.9	0.902	ND	ND	ND
PL	198(165–237)	216(181–254)	0.012^*^	ND	ND	ND
PT	10.9(10.3–11.5)	10.8(10.3–11.4)	0.198	11(10.5–11.7)	10.7(10.3–11.3)	0.002^*^
APTT	25.6(24.3–27.3)	26.3(24.3–28)	0.221	25.45(24.3–27.2)	26.1(24.4–27.9)	0.139
ALB	3.75(3.4–4.09)	3.79(3.45–4.09)	0.501	ND	ND	ND
LDL	110(92–131)	114(95–137)	0.23	ND	ND	ND
HDL	43.5(37.65–52)	40(35–51)	0.064	ND	ND	ND
TCHO	178.5(157–203)	183(158–206)	0.353	ND	ND	ND
TG	94.5(74.5–140.5)	109(83–154)	0.034^*^	ND	ND	ND
BUN	14.75(12–20)	12.05(9.55–16.85)	0.001^*^	ND	ND	ND
UA	5.42 ± 1.73	5.68 ± 1.49	0.187	ND	ND	ND
AST	28(21–37)	24(19–29)	<0.001^*^	ND	ND	ND
ALT	23(17–32)	21(17–30)	0.133	ND	ND	ND
Cr	0.95(0.8–1.17)	0.9(0.76–1.1)	0.125	ND	ND	ND
Glucose	128(109–163)	119.5(99–148)	0.032^*^	ND	ND	ND
Hb1	6(5.7–6.9)	5.8(5.6–6.3)	0.053	ND	ND	ND
CRP	17.1(5.59–61.16)	9.07(3.09–30.78)	0.007^*^	ND	ND	ND
Na	139(137.4–141)	138.85(137–140)	0.162	ND	ND	ND
K	3.7(3.5–4)	3.7(3.31–3.94)	0.231	ND	ND	ND
Segment	60.76 ± 12.46	61.54 ± 12.14	0.564	ND	ND	ND
Lymphocyte	30.15 ± 11.56	30.05 ± 10.61	0.934	ND	ND	ND
SPG	1.02(1.01–1.02)	1.01(1.01–1.02)	<0.001^*^	1.02(1.01–1.02)	1.01(1.01–1.02)	<0.001^*^
SBP	150(133–162)	143.5(129–157)	0.031^*^	150(133–162)	146(130–158)	0.098
NIHSS-Entry	14(9–19)	8.5(6–13)	<0.001^*^	ND	ND	ND
NIHSS-Discharge	10(6–17)	2(0–4)	<0.001^*^	ND	ND	ND
Complication						
Infection			<0.001^*^			<0.001^*^
No	156(62.4%)	119(90.15%)		116(58.59%)	159(86.41%)	
Yes	94(37.6%)	13(9.85%)		82(41.41%)	25(13.59%)	
Hemorrhage			<0.001^*^			0.004^*^
No	226(90.4%)	131(99.24%)		178(89.9%)	179(97.28%)	
Yes	24(9.6%)	1(0.76%)		20(10.1%)	5(2.72%)	
Gout			0.378			0.973
No	233(93.2%)	126(95.45%)		186(93.94%)	173(94.02%)	
Yes	17(6.8%)	6(4.55%)		12(6.06%)	11(5.98%)	
Proteinuria			<0.001^*^			<0.001^*^
No	116(53.21%)	78(75%)		82(48.24%)	112(73.68%)	
Yes	102(46.79%)	26(25%)		88(51.76%)	40(26.32%)	
Bun/Cr			0.012^*^			0.101
<15--Non-dehydration	100(42.74%)	64(57.14%)		82(43.39%)	82(52.23%)	
≥15--Dehydration	134(57.26%)	48(42.86%)		107(56.61%)	75(47.77%)	
Toast Dx			0.001^*^			
Cardioembolism	94(37.6%)	33(25%)		ND	ND	
Lacune	30(12%)	39(29.55%)		ND	ND	
Large-artery atherosclerosis	56(22.4%)	24(18.18%)		ND	ND	
Other determined	3(1.2%)	1(0.76%)		ND	ND	
Undetermined	57(22.8%)	30(22.73%)		ND	ND	

Good prognosis was defined as mRS ≤ 2 and BI >60. Bad prognosis was defined as mRS > 2 and BI ≤60

*Abbreviations*: *BI* Barthel Index, *mRS* modified rankin scale, *ND* not determined. For all other abbreviation see the List of Abbreviations

* *p* < 0.05, significantly different between bad and good prognosis by mRS or BI

We also determined that subjects with an unfavorable prognosis differed significantly from those with a favorable prognosis in several clinical variables (Table 2). However, these variables were not completely overlapping when subjects were classified with the mRS (bad prognosis: 250 of 382 subjects) or the BI (bad prognosis: 198 of 382 subjects). For both scales, mean age, days of admission, urine specific gravity (SPG), and rates of infection (both *p* < 0.001), hemorrhage (mRS: *p* < 0.001; BI: *p* = 0.004), and proteinuria (both *p* < 0.001) were higher for those with an unfavorable prognosis than for those with a favorable one. Subjects with a bad prognosis based on BI score had higher prothrombin time, while use of the mRS indicated that levels of BUN, aspartate aminotransferase (AST), glucose, C reactive protein (CRP), SBP, and scores on the NIHSS at entry and discharge were higher among those with a bad prognosis. Use of the mRS also indicated that subjects with a score greater than 2 had lower scores on the Glasgow Coma Scale (GCS) and lower levels of platelets (PL) and TG; however, none of these variables differed significantly by prognosis when subjects were classified with the BI. Significant differences were also found between prognosis and stroke subtype, as stratified by the TOAST classification system, when the mRS was used to assess disability (*p* = 0.001) (Table 2).

### Identification of variables associated with prognosis

To analyze the associations between clinical characteristics and prognosis, we conducted multivariate analyses, with the variables listed in Table 2 as independent variables and a favorable prognosis — as assessed by the mRS and BI — as dependent variables. Multivariable logistic regression with a forward stepwise method revealed that dehydration was not significantly associated with unfavorable mRS or BI scores. Scores on the mRS were associated with levels of platelets (PL) and urine specific gravity (SPG); scores on the BI were associated with gender, infection, and proteinuria (data not shown).

Although dehydration was not a significant variable in the stepwise regression, we entered this parameter in the multivariate models. Accordingly, dehydration, PL, and SPG were included in a multivariable model of prognosis assessed with the mRS, and dehydration, gender, infection, and proteinuria were included in the model of prognosis as judged by the BI. After adjusting for the variables mentioned above, our analysis revealed that subjects with dehydration had significantly lower odds of achieving a favorable score on the mRS (≤2) than did those without dehydration (OR = 0.46, *p* = 0.004; Table 3). Dehydration also significantly lowered the odds of achieving a favorable score on the BI (≥60; OR = 0.58, *p* = 0.041; Table 4). Despite these findings, dehydration did not increase the risk of an unfavorable score on either scale when the subjects were stratified into the stroke subtypes described in the TOAST classification system (cardioembolism, lacune, and large-artery atherosclerosis; *p* > 0.05 for both; Tables 3 and 4).

**Table 3:** Multivariate analysis of variables associated with prognosis as assessed by the mRS

Variable	OR (95% CI)	*p*-value
Multivariate analysis after the forward stepwise method		
Bun/Cr		
< 15--Non-dehydration	ref	
≥ 15--Dehydration	0.46(0.27–0.79)	0.004^*^
PL	1.003(0.9996–1.01)	0.079
SPG	1.28 × 10^−31^ (4.88 × 10^−49^ - 3.38 × 10^−14^)	0.001^*^
Stratified by TOAST		
For Cardioembolism		
Bun/Cr		
< 15--Non-dehydration	ref	
≥ 15--Dehydration	0.96(0.37–2.46)	0.929
PL	0.999(0.99–1.01)	0.766
SPG	7.71 × 10^−25^(8.76 × 10^−52^ - 677.77)	0.079
For Lacune		
Bun/Cr		
< 15--Non-dehydration	ref	
≥ 15--Dehydration	0.24(0.06–1.01)	0.052
PL	1.02(1.004–1.04)	0.019^*^
SPG	9.75 × 10^−78^(5.11 × 10^−163^ - 1.86 × 10^8^)	0.077
For Large-artery atherosclerosis		
Bun/Cr		
< 15--Non-dehydration	ref	
≥ 15--Dehydration	0.65(0.20–2.08)	0.472
PL	1.004(0.997–1.01)	0.261
SPG	1.38 × 10^−25^ (9.05 × 10^−62^ - 2.10 × 10^11^)	0.178

*Abbreviations*: *BI* Barthel Index, *BUN* blood urea nitrogen, *Cr* creatinine, *mRS* modified Rankin Scale. For all other abbreviations see the List of Abbreviations

* *p* < 0.05, significantly associated with prognosis

**Table 4:** Multivariate analysis of variables associated with prognosis as assessed by the BI

	OR (95% CI)	*p*-value
Multivariate analysis after the forward stepwise method		
Bun/Cr		
< 15--Non-dehydration	ref	
≥ 15--Dehydration	0.58(0.35–0.98)	0.041^*^
Sex		
Female	ref	
Male	1.59(0.92–2.77)	0.097
Infection		
No	ref	
Yes	0.34(0.19–0.62)	<0.001^*^
Proteinuria		
No	ref	
Yes	0.42(0.25–0.71)	0.001^*^
Stratified by TOAST		
For Cardioembolism		
Bun/Cr		
< 15--Non-dehydration	ref	
≥ 15--Dehydration	0.84(0.29–2.41)	0.743
Sex		
Female	ref	
Male	3.31(1.22–8.98)	0.019^*^
Infection		
No	ref	
Yes	0.39(0.13–1.16)	0.091
Proteinuria		
No	ref	
Yes	0.15(0.05–0.43)	<0.001^*^
For Lacune		
Bun/Cr		
< 15--Non-dehydration	ref	
≥ 15--Dehydration	0.63(0.18–2.18)	0.470
Sex		
Female	ref	
Male	0.82(0.23–2.93)	0.755
Infection		
No	ref	
Yes	0.76(0.16–3.48)	0.720
Proteinuria		
No	ref	
Yes	1.29(0.34–4.92)	0.708
For Large-artery atherosclerosis		
Bun/Cr		
< 15--Non-dehydration	ref	
≥ 15--Dehydration	0.61(0.2–1.91)	0.397
Sex		
Female	ref	
Male	0.93(0.23–3.8)	0.914
Infection		
No	ref	
Yes	0.23(0.06–0.92)	0.038^*^
Proteinuria		
No	ref	
Yes	1.08(0.31–3.82)	0.905

*Abbreviations*: *BI* Barthel Index, *BUN* blood urea nitrogen, *Cr* creatinine, *mRS* modified Rankin Scale. For all other abbreviations see the List of Abbreviations

* *p* < 0.05, significantly associated with prognosis

Multivariate analysis of the remaining clinical variables identified in the stepwise regression of prognosis revealed that several remained as significant predictors of achieving a favorable score on the mRS or the BI. For the mRS, favorable scores were negatively correlated with levels of SPG (OR = 1.28 × 10^−31^, *p* = 0.001; Table 3). Upon stratification by TOAST subtype, levels of PL remained significant for the subtype of lacune, but levels of SPG were significant for none of them (Table 3). For the BI, the odds of achieving a favorable score decreased with the presence of an infection (OR = 0.34, *p* < 0.001) or proteinuria (OR = 0.42, *p* = 0.001; Table 4). When stratified by TOAST subtype, gender and rate of proteinuria were independent predictors of outcome for patients with cardioembolism. In addition, the rate of infection was a significant predictor of outcome for patients with large-artery atherosclerosis (Table 4).

## Discussion

Dehydration is a common problem among patients admitted for IS, and dehydrated patients are at increased risk of mortality and poor outcomes. While the risks of dehydration are well established, data are lacking regarding whether or not thrombolytic therapy modifies that risk. This study is the first to determine if dehydration remained a negative prognostic factor for the outcomes of patients with IS who were treated with tPA. We determined that an increased BUN/Cr ratio was associated with a bad prognosis at discharge as assessed by both the mRS and the BI. These findings have important clinical ramifications, as current guidelines lack recommendations for evaluating prognosis when a patient leaves the hospital [27]. We also determined that women were more likely than men to be dehydrated. Taken together, our results serve as an important update to the field and indicate that use of thrombolytic therapy does not eliminate the need to closely monitor hydration status in patients with IS, particularly women.

Despite the fact that thrombolytic therapy is a crucial aspect of stroke management, no other study has yet examined the risk of dehydration among patients with IS who receive tPA. Two of the earliest studies to link dehydration to poor outcomes after stroke, those of Bhalla et al. in 2000 and Kelly et al. in 2004, did not report if tPA or other thrombolytics were administered to their subjects [15, 16]. At the time these studies were reported, it had been less than 10 years since tPA had been approved by the United States Food and Drug Administration and thrombolytic therapy had been incorporated into the guidelines of the American Heart Association (AHA) [43]. From 2005 onwards, almost all of the studies that were conducted reported information regarding the use of thrombolytic therapy; however, non-uniform use and exclusion of patients who received tPA prevented analysis of the impact on the risk of dehydration. This study makes the important discovery that use of tPA or other fibrinolytic agents does not improve recovery so much as to remove the negative influence of dehydration on prognosis.

The confirmatory results obtained by using both the mRS and the BI indicate that these scales are equally reliable tools to assess prognosis. We chose a cut off value of ≤2 for the MRS and ≥60 for the BI because both are recognized as representing a state of independence [34, 41, 44, 45], and both are routinely used in published reports of stroke trials, either separately or together [33, 34]. Despite their widespread use, Uyttenboogaart et al. have suggested that these cut-off values may not be optimized and that an mRS score of 2 is better matched with a score of 90 on the BI [46]. Although our data do not support this conclusion, future research should re-examine the prognostic strength of dehydration by assessing outcomes with additional mRS and BI cut-off scores.

Our finding that dehydration remains a negative prognostic factor for patients receiving tPA after an IS reinforces the conclusions of previous studies that assessed a diversity of outcomes but did not account for the use of thrombolytic therapy. Previous studies from our institution have demonstrated that collateral blood flow around the middle cerebral artery does not develop as robustly in dehydrated patients as it does in well-hydrated patients, when assessed with magnetic resonance imaging at 3 days post stroke [20]. We have also generated substantial data that show an increased risk of stroke in evolution within a 3-day window when dehydration was assessed by either the BUN/Cr ratio or by urine specific gravity [21, 23–25]. Dehydrated patients have routinely been found to be at higher risk of a poor outcome when endpoints were focused on later time points as well. Kelly et al. examined the impact of dehydration on the occurrence of venous thromboembolism [16], Rowat and colleagues examined the rates of mortality and dependence at discharge, and members of our group have examined infection rate, length of stay in the hospital, and mRS and BI scores at discharge [17, 22, 26]. For all of these, poor hydration is associated with a poor outcome. Finally, studies by Bhalla et al. and Schrock et al. indicate that dehydration while in the hospital increases risk of death or dependence at 1 or 3 months after discharge [15, 18]. Because this study only assessed mRS and BI scores at discharge, future studies should confirm that use of tPA does not reverse the negative influence of dehydration on these additional indicators of recovery.

This analysis identified several clinical characteristics with a possible relationship to dehydration or a poor prognosis. Parameters that were over-represented in dehydrated patients included female gender, age, and levels of hemoglobin and triglycerides. Few studies of the effects of dehydration have monitored triglycerides and hemoglobin, but these should be evaluated in the future. Other studies did report data for age and gender, and the results are mixed. Among two of our own studies [20, 22], only one found that dehydration was associated with age and female gender [22]. The study that did not find over-representation of older patients and women in the dehydrated group, by Chang et al., was a more specialized study that only enrolled patients who received MRI within 3 days of admission and were diagnosed with an occlusion of the middle cerebral artery [20]. Another more generalized study, by Rowat et al., also found that age and gender ratios differed significantly among the hydrated and dehydrated groups [17]. At present, we have not determined why women or the elderly would be more likely to be dehydrated when presenting with an IS; physiological and non-physiological factors may contribute. As dehydration is a strong predictor of outcome, more research is needed to shed light on this issue. It should be mentioned that women may benefit more from tPA treatment than do men [47–50]; therefore, the unfavorable prognosis of women who are dehydrated may not necessarily translate into inferior outcomes if efforts are made to ensure that they receive timely thrombolytic therapy.

In addition to evaluating the ability of dehydration to predict an unfavorable outcome, our study also sought to identify other clinical variables with predictive significance. As with dehydration, the mRS and BI identified a non-overlapping set of clinical variables. These variables also differed in how robust they were to stratification by TOAST subtype classification. When judging prognosis by the mRS, only platelet level remained as an independent predictor of outcome for any of the 3 TOAST subtypes. When the BI was used to assess prognosis, complex patterns emerged for associations between prognosis and subtype: the rate of proteinuria remained as significant predictors of outcome for patients who experienced a cardioembolism, and gender gained significance. However, for the subtype of lacune, no clinical factor remained as an independent predictor. For patients with large-artery atherosclerosis, the rate of infection was the only independent predictor of outcome. These data may not be due to inherent differences in outcome for patients with these types of stroke, as no clear consensus has emerged that any of them are associated with greater mortality [51–54]. Additional studies should be conducted to confirm that prognosis of different stroke types is predicted by distinct clinical characteristics.

The study had several limitations. First, the prognostic significance of dehydration for long term-outcomes remains unknown, as the primary endpoint of this study was evaluated at the time of discharge from the hospital. Second, there may be some deviation in the results because the hospitalization times were not uniform among the patients. Finally, a minority of patients may have been well hydrated but still had a Bun/Cr ratio of 15 or more. Although we estimate that this applies to only 30% of subjects, our results could have been influenced by this variability among patients in the dehydration group.

## Conclusions

We have conducted a retrospective study to assess the ability of dehydration to predict the outcome of patients with IS receiving tPA. As far as we know, this study is the first to examine this issue and thus makes an important contribution to the field. Our results indicate that dehydration remains a negative prognostic indicator for outcome at discharge. Accordingly, we recommend that hydration status should continue to be routinely monitored for all patients with IS, and closely monitored in women and older patients, regardless of the use of thrombolytic therapy.

## Abbreviations

ALB: Albumin
ALT: Alanine aminotransferase
APTT: Activated partial thromboplastin time
AST: Aspartate aminotransferase
BI: Barthel Index
BUN: Blood urea nitrogen
Cr: Creatinine
CRP: C reactive protein
GCS: Glasgow coma scale
Glucose: Blood glucose
Hb: Hemoglobin
HbA1c: Glycosylated hemoglobin
Hct: Hematocrit
HDL: High-density lipoprotein
K: Potassium
LDL: Low-density lipoprotein
mRS: modified Rankin Scale
Na: Sodium
ND: Not determined
NIHSS: National Institutes of Health Stroke Scale
PL: Platelets
PT: Prothrombin time
SBP: Systolic blood pressure
SPG: Urine specific gravity
TCHO: Total cholesterol
TG: Triglyceride
TOAST: Trial of Org 10,172 in Acute Stroke Treatment
tPA: tissue plasminogen activator
UA: Uric acid
WBC: White blood cell count
